# Utilizing random Forest QSAR models with optimized parameters for target identification and its application to target-fishing server

**DOI:** 10.1186/s12859-017-1960-x

**Published:** 2017-12-28

**Authors:** Kyoungyeul Lee, Minho Lee, Dongsup Kim

**Affiliations:** 1 Department of Bio and Brain Engineering, Korea Advanced Institute of Science and Technology, 291, Daehak-ro, Yuseong-gu, Daejeon, 34141 Republic of Korea; 2 Catholic Precision Medicine Research Center, College of Medicine, The Catholic University of Korea, 222, Banpo-daero, Seocho-gu, Seoul, 06591 Republic of Korea

**Keywords:** Virtual screening, Target identification, SAR modeling, Random forest, Extended connectivity fingerprint, Target fishing server

## Abstract

**Background:**

The identification of target molecules is important for understanding the mechanism of “target deconvolution” in phenotypic screening and “polypharmacology” of drugs. Because conventional methods of identifying targets require time and cost, *in-silico* target identification has been considered an alternative solution. One of the well-known *in-silico* methods of identifying targets involves structure activity relationships (SARs). SARs have advantages such as low computational cost and high feasibility; however, the data dependency in the SAR approach causes imbalance of active data and ambiguity of inactive data throughout targets.

**Results:**

We developed a ligand-based virtual screening model comprising 1121 target SAR models built using a random forest algorithm. The performance of each target model was tested by employing the ROC curve and the mean score using an internal five-fold cross validation. Moreover, recall rates for top-*k* targets were calculated to assess the performance of target ranking. A benchmark model using an optimized sampling method and parameters was examined via external validation set. The result shows recall rates of 67.6% and 73.9% for top-11 (1% of the total targets) and top-33, respectively. We provide a website for users to search the top-*k* targets for query ligands available publicly at http://rfqsar.kaist.ac.kr.

**Conclusions:**

The target models that we built can be used for both predicting the activity of ligands toward each target and ranking candidate targets for a query ligand using a unified scoring scheme. The scores are additionally fitted to the probability so that users can estimate how likely a ligand–target interaction is active. The user interface of our web site is user friendly and intuitive, offering useful information and cross references.

**Electronic supplementary material:**

The online version of this article (10.1186/s12859-017-1960-x) contains supplementary material, which is available to authorized users.

## Background

Toxicity, low efficacy, and uncertain clinical safety of novel drugs are the main causes of clinical failure, thus increasing the cost and time to develop novel approved drugs [1]. Many researchers anticipate that a network-based approach might improve the efficiency of drug discovery [2–4]. Recent advancements in the field of phenotypic screening are providing new insights for the chemical response of biological networks or systems [5]. However, a “target deconvolution,” wherein the actual targets of the molecules are disclosed, is crucial in understanding the mechanism of action, which remains challenging [6]. On the other hand, even if the target of a drug is already known, it is still necessary to predict the association with other targets. The term “polypharmacology” is broadly defined as the trait of pharmaceutical agents to interact with multiple targets or pathways. It is generally perceived that most drugs act on more than one target [7]. Discovering polypharmacology of drugs can be useful not only for drug repositioning to determine novel ways to facilitate drugs but also for predicting side effects to avoid harmful responses beforehand [8–10].

Conventional methods of identifying molecular targets include affinity chromatography, 2D gel electrophoresis, and other methods based on the mRNA expression [11, 12]. Although these methods can be used to identify molecular targets with good accuracy, the time and cost of such in-vitro assays make it difficult to test large ligand–target interactions [13]. Because of these limitations, *in-silico* target prediction is considered a promising alternative for target identification. The *in-silico* target prediction can be classified into two categories based on the type of data to be used: 1) ligand-based method, and 2) structure-based method [14]. In particular, the ligand-based methods are advantageous in large-scale virtual screening because of the low computational cost and high feasibility [15]. One of the most popular methods of ligand-based target identification involves classifying the ligands using structure-activity relationships (SARs). Various machine-learning techniques have been applied in this field including support-vector machine (SVM), naïve Bayesian classifier (NB), artificial neural network (ANN), and kernel discrimination [16]. Among those methods, NB is known to be effective for target classification of ligands, but weak for the cases when molecular features have conditional dependencies [15]. Other machine-learning methods have not successfully applied for finding true targets of drug-like molecules from large scale (~1000) protein database as the extent as we know. We chose random forest (RF) algorithm [17] which is an ensemble of decision trees because it is believed to avoid overfitting and deal with imbalanced classes properly.

The principle behind the SAR approach is that structurally similar ligands might have similar properties [18]. The objective is searching a chemical space comprising ligand structures with known activities to predict the activity of a query ligand. In the *in-silico* target prediction, structures of ligands can be represented as molecular descriptors such as fingerprints, and the activity can be defined as the binding with specific targets. The algorithms developed for this purpose are generally used to build a target-classification model [19–21] using binding-activity data obtained from diverse chemogenomics libraries such as PubChem [22], ChEMBL [23], WOMBAT [24], and ZINC [25]. The model derived from this process represents key structural properties of molecules that aid in binding with the targets. Thereafter, the ranks of the targets for a query ligand are estimated based on the scores of the model. A few web servers [20, 26, 27] were recently developed to provide top-rank targets of the query ligand that users submit in SMILES format or draw using MarvinSketch [28].

**Fig. 1:**
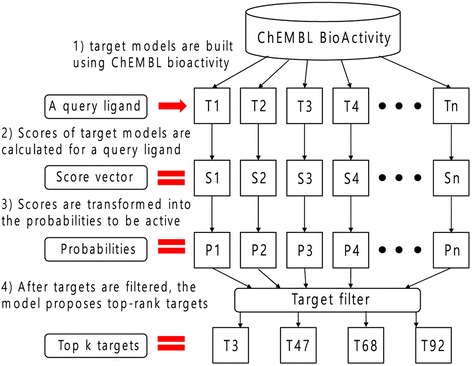
Overall process of RF-QSAR. First of all, 1121 target models are built by bioactivity data from ChEMBL database. As a user input a query ligand to the server, scores for target models are calculated to build a score vector. Then, the score vector is transformed into the probabilities to be active. Finally, top-*k* targets are proposed ranked by their probabilities to the query ligand. Targets to search can be filtered by their classes according to user’s preference

Some issues regarding the use of SARs for target prediction include imbalance in the amount of active data and ambiguity of inactive ligands throughout targets. These problems are based on the dependency of ligand-based approaches on the available data [16]. Major proteins, which are actively experimented for decades, have more active data than other targets. Furthermore, in many related studies, ligands that are not known to be active for a target are considered inactive ligands for the target [13, 20, 26]. However, some of the actual ligand–target interactions might not have been experimented. Such a bias observed in the database can lead to a failure in predicting the true interactions, particularly for targets with less active data. In this study, the objective is to overcome such bias by building multiple target models using random forest algorithm with a standardized sampling method. In particular, based on the cross-validation results, the standard to define inactive ligands and the ratio between the active and inactive ligands were optimized. Hence, we built a comprehensive model comprising multiple target models. The model is applicable for two types of usage: 1) predicting the activity of ligands toward each target 2) target prediction of a query ligand by comparing the results from the individual models. The completed model is provided through a free accessible target-fishing server at http://rfqsar.kaist.ac.kr. Figure 1 depicts the overall process of the server.

### Methods

#### Data collection from the chemogenomics database

**Fig. 2:**
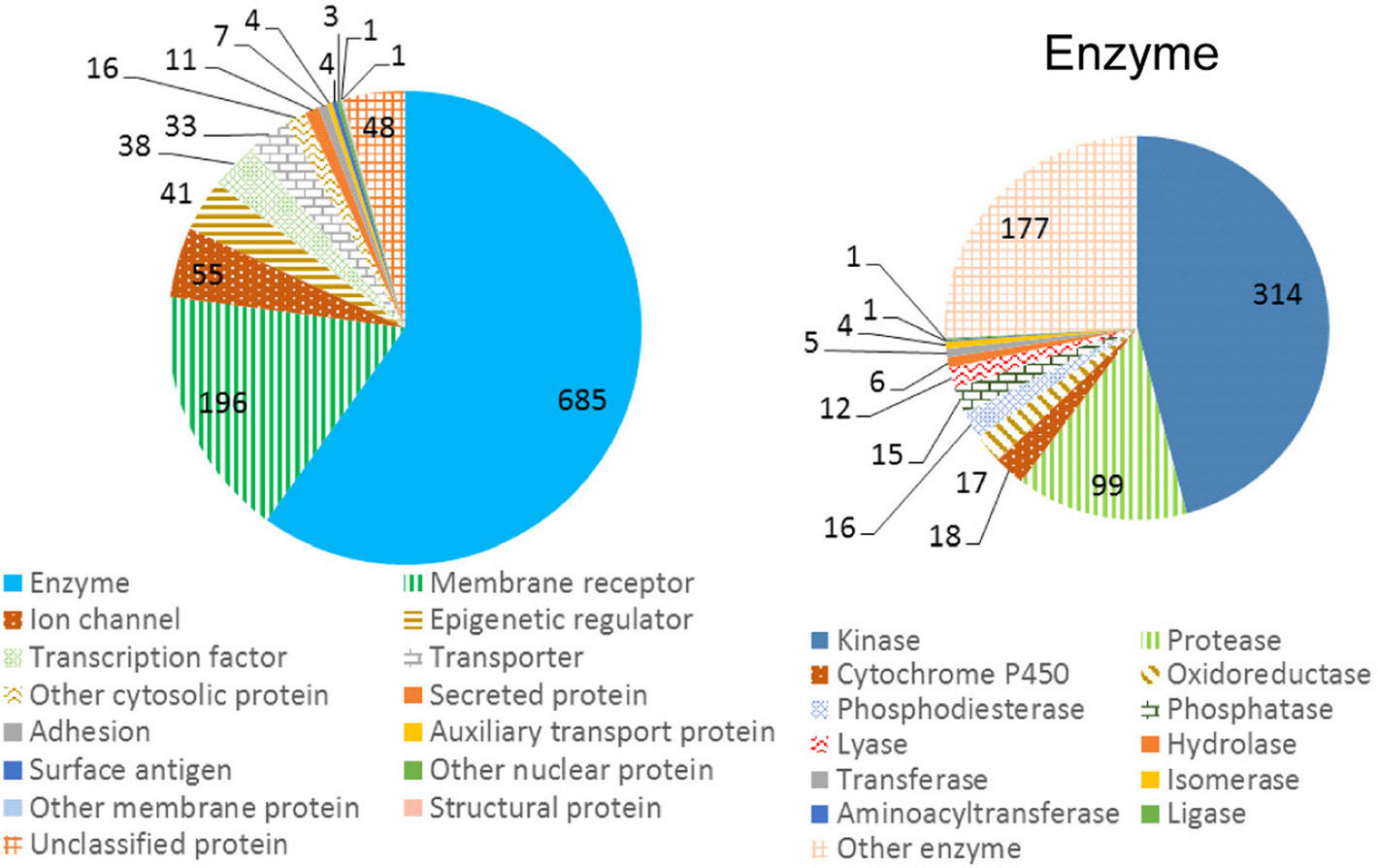
The class distribution of the target models. Since the majority of targets are enzymes, enzymes are further classified by the enzyme subclass such as kinase, protease, and phosphatase. The total sum of the number of targets for each class is 1143 instead of 1121 (the total number of targets) because a few targets belong to multiple classes

In this study, ChEMBL (Version 20) database [23] was used to build the active and inactive training datasets for modeling the SARs. The active ligands for specific targets were defined as molecules with activities lower than 10 μM tested using IC50, EC50, Ki, and Kd [13, 20, 27, 29]. Among the human proteins deposited in the ChEMBL, proteins with at least 10 known binding ligands were selected for developing the models to avoid unreliable models with insufficiently low amount of activity data. The selected training set corresponds to 1121 targets and 235,713 unique ligands with the number ranging from 10 to 4305 of known active ligands for each target. Moreover, target information including class, sequence, and domains are retrieved from the ChEMBL database for further utilization in the server. The 1121 targets were classified under various target classes including enzymes, membrane receptor, ion channel, etc. As most of the targets (685) were enzymes, they were further classified by enzyme subclass such as kinase, protease, and phosphatase. Figure 2 shows the class distribution of the target models. The detailed MySQL commands used to extract bioactivities from the ChEMBL can be obtained from Additional file 1.

#### Model building using random Forest algorithm

The ligand data obtained from ChEMBL were standardized using ChemAxon standardizer [30] with options “Remove Fragment,” “Neutralize,” “Remove Explicit Hydrogens,” “Clean 2D,” “Mesomerize,” and “Tautomerize.” The resulting SMILES were used to generate ECFP_4 fingerprints (extended-connectivity fingerprints with diameter of 4) with 2048-bit length string using RDKit python module [31]. Subsequently, for each target, the ligands with known active data were used as positive ligands whereas the ligands without active data were assumed as negative (inactive) ligands. After the sampling and filtering processes described below, the target models were trained based on the fingerprint data of active and inactive ligands using a random forest algorithm implemented in the sklearn python module [32]. We constructed an individual model for each target to be used for both activity prediction and target fishing. The random-forest algorithm is known to reduce the bias due to overfitting and class imbalance. Because the bioactivity data obtained from ChEMBL have several class imbalances between the active and the inactive data and even between the targets, random-forest classification method may be able to handle such a bias effectively. Random forest algorithm applies bagging and subset selection techniques to overcome the instability of decision tree model caused by its hierarchical nature. Multiple training sets are randomly sampled to build multiple trees and the features are refined based on out-of-bag cases [15]. The number of trees for each target model is set to 100 in this study. The score, ranging from 0 to 1, is defined as the proportion of trees which decide a query ligand is active.

### Data preprocess before training

Before training the models, several data preprocessing steps were conducted to deal with class imbalance and ambiguity in the inactive data. For a few targets, the ratio of the active ligands to the inactive ligands is as large as 1:23,570, indicating that the number of active ligands is considerably smaller than that of the inactive ligands. Because such an imbalance can lead to a significant reduction in the accuracy, two different sampling methods were employed to handle the class imbalance. A negative-undersampling method was used to randomly select only a subset of the inactive ligands until the ratio reaches to a particular value. A positive-oversampling method was used to repeatedly select the active ligands [33]. Because of practicality, the positive-oversampling method was performed by imposing larger weights on the active ligands when trained. In this study, we employed a common ratio across the targets to avoid overfitting the targets with a large number of active ligands. Defining the inactive ligands is often controversial as the inactive ligands are relatively ambiguous compared to the active ligands. Some ligands without the activity data might be actually active, which should be excluded from the set of inactive ligands. By calculating the Tanimoto coefficient (Tc) similarity between the fingerprints, ligands having similar active data with a particular threshold were excluded from the inactive ligands [29].

### Internal cross validation

To validate the performance of the random forest models, prediction performances of the models were evaluated for the training data using a five-fold cross-validation method. 235,713 active ligands across all the targets were divided into five subsets and one subset was set aside as a test set. The rest of the ligands were used as the training data to develop the models followed by the data preprocess. The scores between the test ligands and the target models were calculated. The ligands with scores higher than the score threshold were then predicted as positive labels and the others were predicted negative. First, the performance of each trained model for the test set was assessed using a receiver-operating characteristic (ROC) curve by varying the score threshold from 0 to 1. In addition, the mean score of the active ligands and that of inactive ligands were compared to check whether the two mean values differ significantly. The ratio between mean score of active ligands and mean score of inactive ligands was computed for each target and averaged by five-fold. Finally, the targets were ranked by ordering the 1121 targets based on their score for each ligand. The Recall was calculated, assuming that the top-*k* values (*k* = 4, 7, 11, 33, 66, 88, and 110) from the ranked list of targets were predicted as positives [13, 29]. The assessments were then averaged over five different test sets. We built and evaluated various target prediction models by changing the sampling methods, ratio between the numbers of inactive and active ligands, and Tc similarity cutoff for the inactive ligands to determine the optimal parameters. Pearson’s chi-squared test was used to evaluate the statistical significance of the difference among parameters when discriminating between true positives and false negatives for the top-11 threshold.

### External validation

Accordingly, a benchmark model using optimized preprocessing method was constructed with the entire training set from ChEMBL version 20. However, an independent validation set was required to evaluate the benchmark model. Hence, we retrieved additional bioactivity data from ChEMBL version 21 and employed them as an external validation set. The external set contains only novel ligands having at least one active target from the target models. The ligands having the same ECFP fingerprints as those in the training set were also removed from the validation set. With the resulting 13,589 external ligands, a score matrix between the validation set and the 1121 target models was obtained. Thereafter, the ROC curve and its area under curve (AUC) value, and the recall for the top-*k* targets (*k* = 11 and 33, which corresponds to 1% and 3% of total targets, respectively) were evaluated and compared with the results obtained in other studies.

### Probability estimation from the model score

**Fig. 3:**
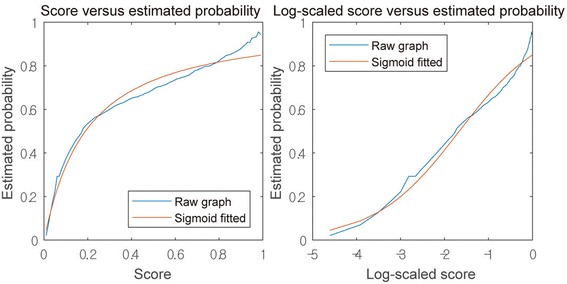
The relationship between model scores and the estimated probabilities of interaction. (Left) A graph of score versus estimated probability. (Right) A graph of log-scaled score versus estimated probability. Estimated probability was fitted to sigmoid function of log-scaled score (Sigmoid fitted)

Although scores of the virtual assay are useful for distinguishing the active ligands from the inactive ligands, users might want to know whether the interactions with the certain scores are in fact active. In case of ranking the targets, some ligands could have low probability of interaction even with high rank targets. To overcome such ambiguity, we propose a probability estimation function to transform the model score into probability of interaction. From the virtual assay of the external set, ligand–target pairs were divided by several score cutoffs ranging from 0 to 1. For each score cutoff, the pairs of the interaction having scores higher than the cutoff were retained. The probability of interaction was estimated based on the number of active pairs divided by the number of total pairs for each cutoff. A graph of log-scaled score versus estimated probability was drawn, and the curve was fitted to the sigmoid function (Additional file 2). Figure 3 shows the graph.

### Web implementation

We implemented our target fishing model to the web based server (http://rfqsar.kaist.ac.kr) so that users can freely search for the predicted targets of the query ligand. Currently, bioactivity data from ChEMBL version 20 was used to build the random forest model with optimized parameters. PHP and jQeury were used for web programming. ChemAxon standardizer [30] is implemented to standardize SMILES format just as used for training. Also, Open Babel software [34] is included to transform ligand structures into 2D figures.

## Results and discussion

### Performance of interval validation

**Fig. 4:**
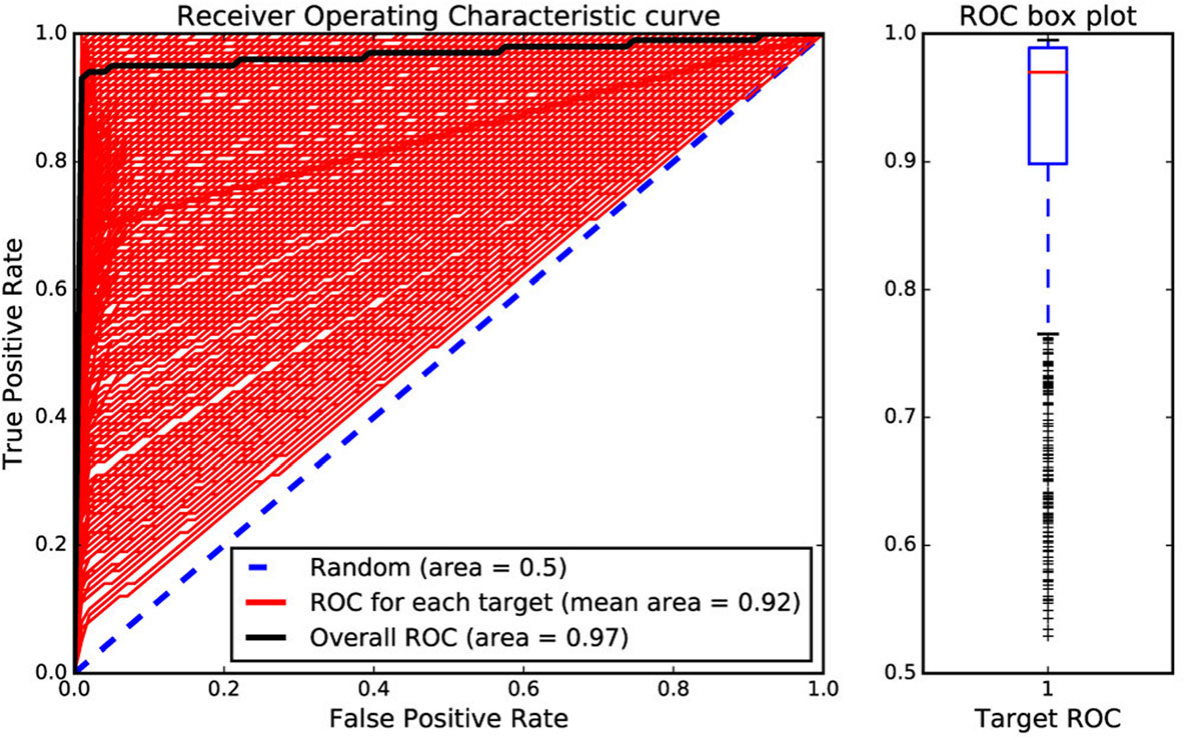
ROC curves and the area under curves computed by internal cross validation. (Left) ROC curves for each target and overall ROC curve. Blue dotted line indicates ROC curve for random selection with AUC = 0.5. Red lines are ROC curves for each target and black line is overall ROC curve built using all the screening data throughout targets. (Right) Box plot of AUC values for targets. Red line indicates the median value of AUC, which is 0.97

**Fig. 5:**
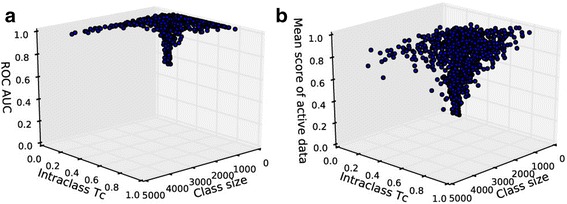
The scatter plot of the performance for each target model versus the model property. Model property includes the number of active ligands (Class size) and the Tc similarity among the active ligands (Intra-class Tc). Each dot on the graph represents the specification of each target model. Overall trend shows models with low performance have small class size and low intra-class Tc. **a** Scatter plot of AUC values. **b** Scatter plot of mean scores of active data

The internal validation of the proposed SAR models was performed using a five-fold cross validation procedure. The performance of the internal validation was measured using the optimized sampling method and parameters. The virtual screening results of the five-fold cross validation were first used to measure the performance for each target model. Hence, the ROC curve for each model was computed by taking the average of the ROC curves from the five folds. The area under the ROC curve (AUC) was evaluated to estimate the performance of each target model. Figure 4 shows the ROC curves for the 1121 target models and boxplot of the AUC values. The overall ROC curve is the curve obtained using the screening data throughout the targets. The AUC value for the overall ROC is 0.97, implying that these models can be used to distinguish the active ligands from the inactive ligands with good sensitivity. The boxplot shows that the AUC values of most of the models (~75%) is above 0.9. Although the AUC values of few models (~7%) are under 0.7, the AUC values of the models are above 0.5 with a median AUC value of 0.97. The models with low AUC value generally have a small number of active ligands (class size) and low Tc similarity among the active ligands (intra-class Tc) as shown in Fig. 5a. This is probably because some of the active ligands to be cross-validated do not have any other active ligands nearby for small and sparse target classes.

**Fig. 6:**
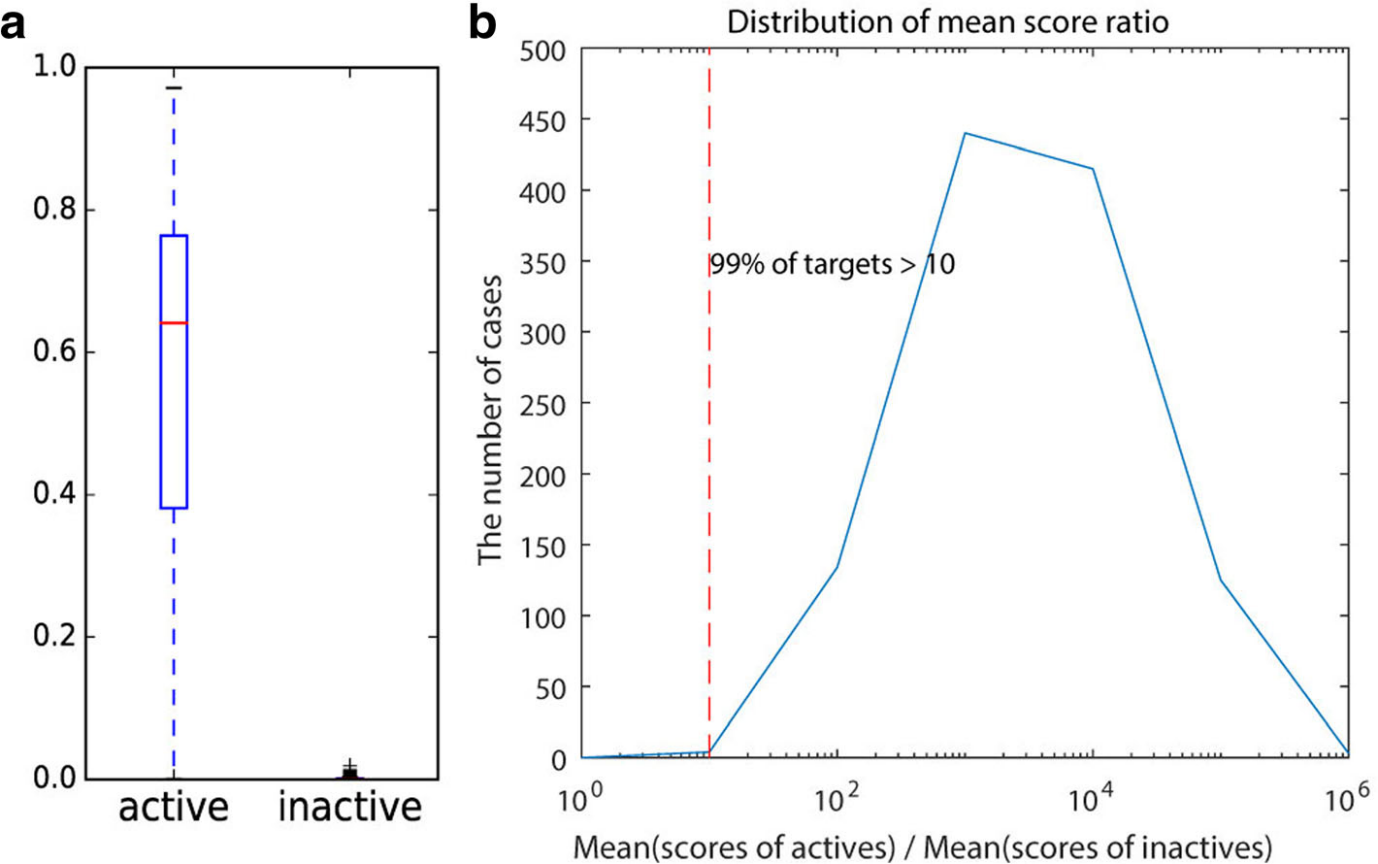
Comparison of the mean scores between the active and inactive ligands for each target. **a** Box plot of the mean scores for the active ligands and inactive ligands. **b** Distribution of the ratio of the mean score of active ligands to the mean score of inactive ligands. Ratio = 10 means that the mean score of active data is 10 times greater than that of inactive ligands for the target. The numbers of targets were measured for the ratio intervals divided by 1, 10, 100, 1000, 10,000, 100,000, 1,000,000 and the x-axis of the graph was log-scaled. The result shows that almost all targets (99%) have the ratio over 10 fold

Because the scores of the target models are to be used to determine the true interaction among many others, the scores of the active ligands should be significantly higher than the scores of the inactive ligands. To verify such trend, the mean scores of the positive and negative sets were calculated for each target using the five-fold cross validation. We observe that the mean score of the negative set is approximately zero for the target models (max = 0.02), whereas the mean score of the positive set is broadly distributed with a median of 0.64 (Fig. 6a). The targets with low mean scores in the positive set generally have small class sizes and low intra-class Tc values, which are similar to the trend observed in the AUC distribution (Fig. 5b). Nevertheless, the mean scores of the positive set of most of the target models (99%) are considerably higher than those of the negative set by at least 10 fold (Fig. 6b).

**Fig. 7:**
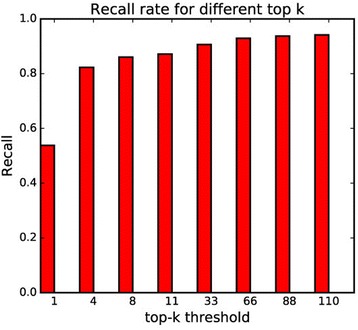
The recall rates for various top *k* values (*k* = 1, 4, 8, 11, 33, 66, 88, 110) measured by internal cross validation. Recall rate is defined as TP / (TP + FN) where TP is True Positive and FN is False Negative. If an active target of a query ligand has rank higher than *k* value, the interaction is counted as TP. Otherwise, it is counted as FN

The virtual screening result for each query ligand is a score vector constructed using the 1121 target models. The main application of our model is ranking the targets for a query ligand so that users are able to obtain a reasonable number of targets to be tested. Hence, the model performance of the target ranking needs to be verified via cross validation. One of the general methods of verifying the performance involves employing the recall rate for the top-rank targets. In this method, the targets ranked in the top-*k* (*k* is the feasible target number) are recognized as active targets for a query ligand, whereas the other targets are assumed inactive. The recall rate is defined as TP / (TP + FN), which is the ratio of the number of detected active targets to the real active targets. The recall rate is averaged over the five different test sets during five-fold cross validation procedure. The higher recall rate means that the sensitivity of the model is better with fewer missing active targets. Figure 7 shows the change in the recall rates for different top-*k* thresholds. The recall rate increases with an increase in the top-*k* threshold. However, if the top-*k* threshold is high, many targets recognized as active might be actually inactive. Moreover, as the number of targets to be checked via experiment increases, the efficiency of the model application decreases. In fact, the recall rate changes only slightly after the top-4 threshold. For practicality, in general, approximately 10 targets out of the total targets are proposed as candidate targets [13, 29, 35]. In our model, the recall rates for the top-4 and top-11 (1% of total targets) targets were 0.823 and 0.871, respectively.

### Parameter optimization

Defining the active and inactive ligands for each target is very important to successfully model the SARs [29, 36]. Two different methods were proposed to build the active and inactive sets for each target model depending on the sampling methods: negative-undersampling and positive-oversampling. The ligands of the targets were sampled until the number of inactive ligands reached a fixed ratio of the number of active ligands (it was set arbitrarily to 20). First, the performances of the different sampling methods were compared by calculating the recall rates for the top 1, 4, 8, and 11 targets and overall AUC value (Table 1). Although the negative-undersampling method slightly outperformed the positives oversampling method in terms of the overall AUC, the recall rate was relatively lower than that obtained using the positive oversampling method. In addition, because the AUC value was sufficiently high in the positive-oversampling method and recall rates are more important for the application of target fishing, we selected the positive-oversampling method as the general sampling method. Positive-oversampling method recognized more active ligands as positives compared to negative-undersampling method with *p*-value = 6.39E-10 for Pearson’s chi-squared test.

**Table 1:** Performance comparison between negative-undersampling and positive-oversampling

Sampling method	Negative-undersampling	Positive-oversampling
Overall ROC AUC	0.975	0.956
Top 1 recall	0.534	0.549
Top 4 recall	0.81	0.822
Top 8 recall	0.849	0.855
Top 11 recall	0.86	0.865

In fact, we built multiple positive-oversampling models with different ratios of the number of inactive ligands to the number of active ligands ranging from 1 to 40. Table 2 presents the performance comparison between the models. The result shows that a balanced ratio between the active and inactive ligands yields the best recall rate in any threshold. The values of the overall AUC follow the same trend. Hence, the ratio of the number of inactive ligands to the number of active ligands was set to one. Pearson’s chi-squared test shows that the model with the ratio of 1 recognized more true positives than those with the ratio of 10, 20, 30, and 40 with p-value of 7.09E-3, 7.60E-4, 6.40E-5, and 1.71E-5 respectively.

**Table 2:** Performance comparison between different ratios of the number of ligands for positive-oversampling

Ratio (inactive/active)	1	10	20	30	40
Overall ROC AUC	0.961	0.956	0.956	0.955	0.955
Top 1 recall	0.549	0.549	0.549	0.549	0.549
Top 4 recall	0.823	0.822	0.822	0.822	0.822
Top 8 recall	0.857	0.856	0.855	0.855	0.855
Top 11 recall	0.868	0.866	0.865	0.865	0.865

**Fig. 8:**
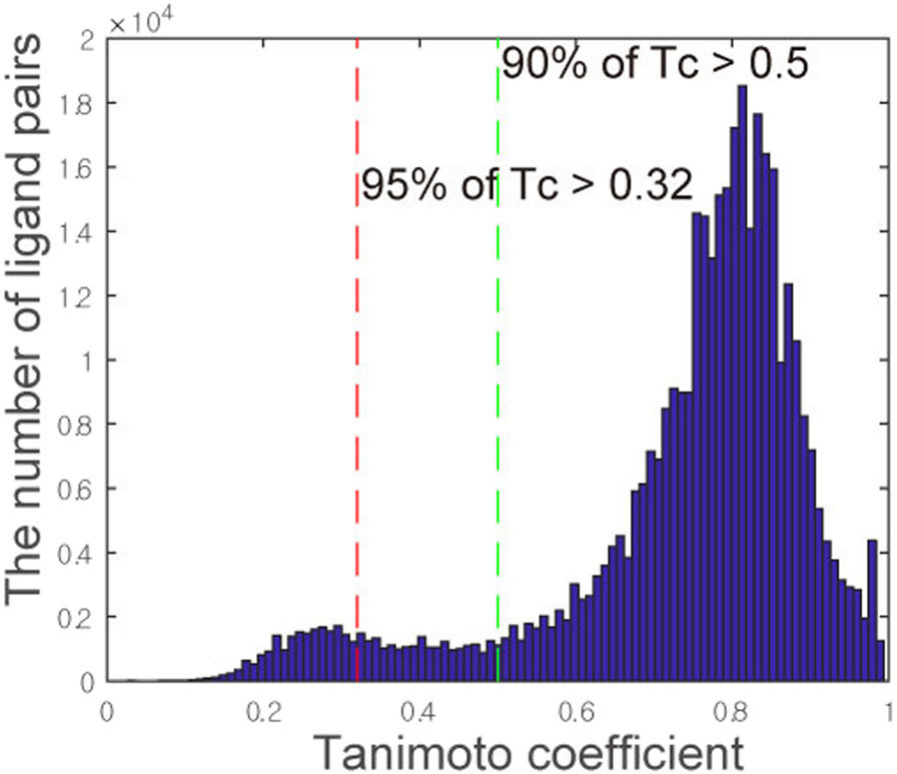
The distribution of Tanimoto coefficients of nearest active ligands for specific targets. The nearest pairs of active ligands in the same targets are collected. The distribution of the Tc values for ligand pairs shows that 90% of Tc values are larger than 0.5 and 95% of Tc values are larger than 0.32 (~ 0.3)

Many inactive ligands used for the target model were not experimentally tested for the target. Some of them would turn out to be active ligands. In particular, the ligands that are similar to known active ligands have higher probability of being active. In some cases, such inactive ligands in the model may cause active queries to be evaluated as inactive. One of the methods of reducing the bias involves excluding the inactive ligands that are similar to active ligands to some extent. The well-known Tc similarity is employed as a cutoff for this purpose. When the Tc similarities between the nearest active ligands within specific targets were examined, 95% of the pairs had Tc similarities above 0.32, and 90% of the pairs had Tc similarities above 0.5 (Fig. 8). For different Tc similarity cutoffs (0.3, 0.5, and w/o cutoff), the recall rates of the target ranking were examined to obtain the best fit for identifying the targets (Table 3). The results obtained by applying the Tc cutoff values showed better performance compared those obtained without the cutoffs. However, the results obtained for Tc cutoffs of 0.3 and 0.5 are somewhat ambiguous. The AUC value increases from a Tc cutoff of 0.3 whereas the recall rates are better for a Tc cutoff of 0.5. We selected a Tc cutoff of 0.5 because, as previously mentioned, the recall rates should be more distinguishable for practicality. The model applying Tc cutoff of 0.5 recognized more true positives compared to that without Tc cutoff with *p*-value of 1.89E-6 for chi-squared test. Accordingly, the benchmark model was built using the positive-oversampling method by employing optimized parameters, such as active/inactive ratio = 1 and Tc cutoff = 0.5.

**Table 3:** Performance comparison between different Tc cutoffs for excluding inactive ligands

Tc cutoff	0.3	0.5	w/o cutoff
Overall ROC AUC	0.973	0.966	0.961
Top 1 recall	0.527	0.538	0.548
Top 4 recall	0.815	0.823	0.823
Top 8 recall	0.858	0.86	0.857
Top 11 recall	0.87	0.871	0.868

### Performance of external validation

**Fig. 9:**
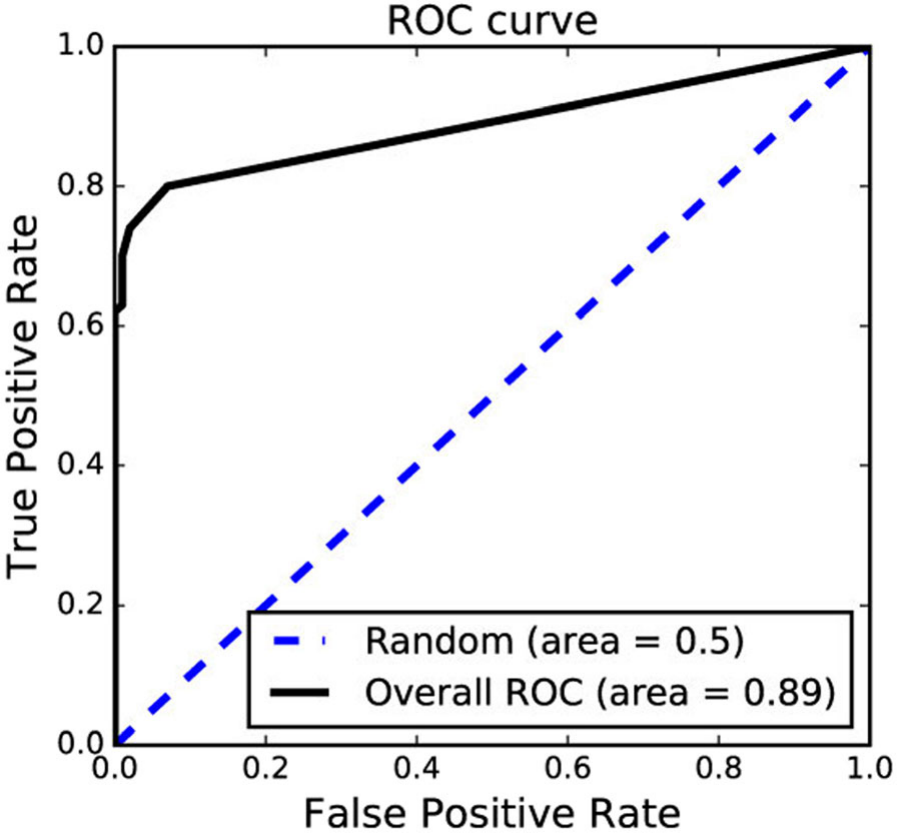
The ROC curve for screening results of the external validation set. 20% of active data and 93% of inactive data from external set have scores of 0, which makes a long straight line at the end of the curve. Active ligand with score of zero might represent novel chemical structures of bioactivity newly discovered by recent experiments

To test the performance of the benchmark model on the novel ligands, an external validation set was developed using the data from new version of ChEMBL. The average Tc similarity value of the external set to the nearest ligands implemented at the benchmark model was 0.55. The virtual-screening result of the external validation set was evaluated using the ROC curve and recall rate. The ROC curve was drawn by defining known active data as positive set, and the area under the ROC curve was 0.89 (Fig. 9). The value is lower compared to the AUC obtained through the cross validation (0.97), largely because a larger population of the active interactions are degraded to score 0. The ROC curve shows that the scores of approximately 20% of the active ligands are zero whereas the scores of 93% of the inactive ligands are zero. Such active ligands with scores of 0 may represent novel chemical structures not explained by the model but included in the external set. Nevertheless, the result indicates that the performance of the benchmark model is still high for external validation with a value of approximately 0.9.

The recall rates for the top-*k* targets were also calculated to verify that the performance of external validation. For the top-11 (1%) targets, the recall rate of the external set using the benchmark model was 67.6%. For the top-33 (3%) targets, the recall rate was 73.9%. This result is slightly better than the performance measured using the Parzen–Rosenblatt Window based Naïve Bayesian model by Alexios Koutsoukas et al., wherein the results were 66.6% and 73.9% for the top 1% and 3% of the targets, respectively [13]. The recall rate obtained using the method proposed in this study is better than that obtained using other naïve Bayesian models such as Laplacian-modified Naïve Bayes (63.3% for top 1% and 72.1% for top 3%) [13] or Bernoulli Naïve Bayes (62.5% for top 1% and 72.5% for top 3%) [29]. While the WOMBAT external set used for these tests has an average Tc value of 0.58 with the training set, the external set used in our test has a value of 0.55, indicating that the difficulty of the problem is increased. Thus, it is fair to say that the performance of current method is better than those of previous methods. Moreover, we expect that the result may be improved by further modification because the current benchmark model is a simple collection of individual target models.

### Target fishing server

**Fig. 10:**
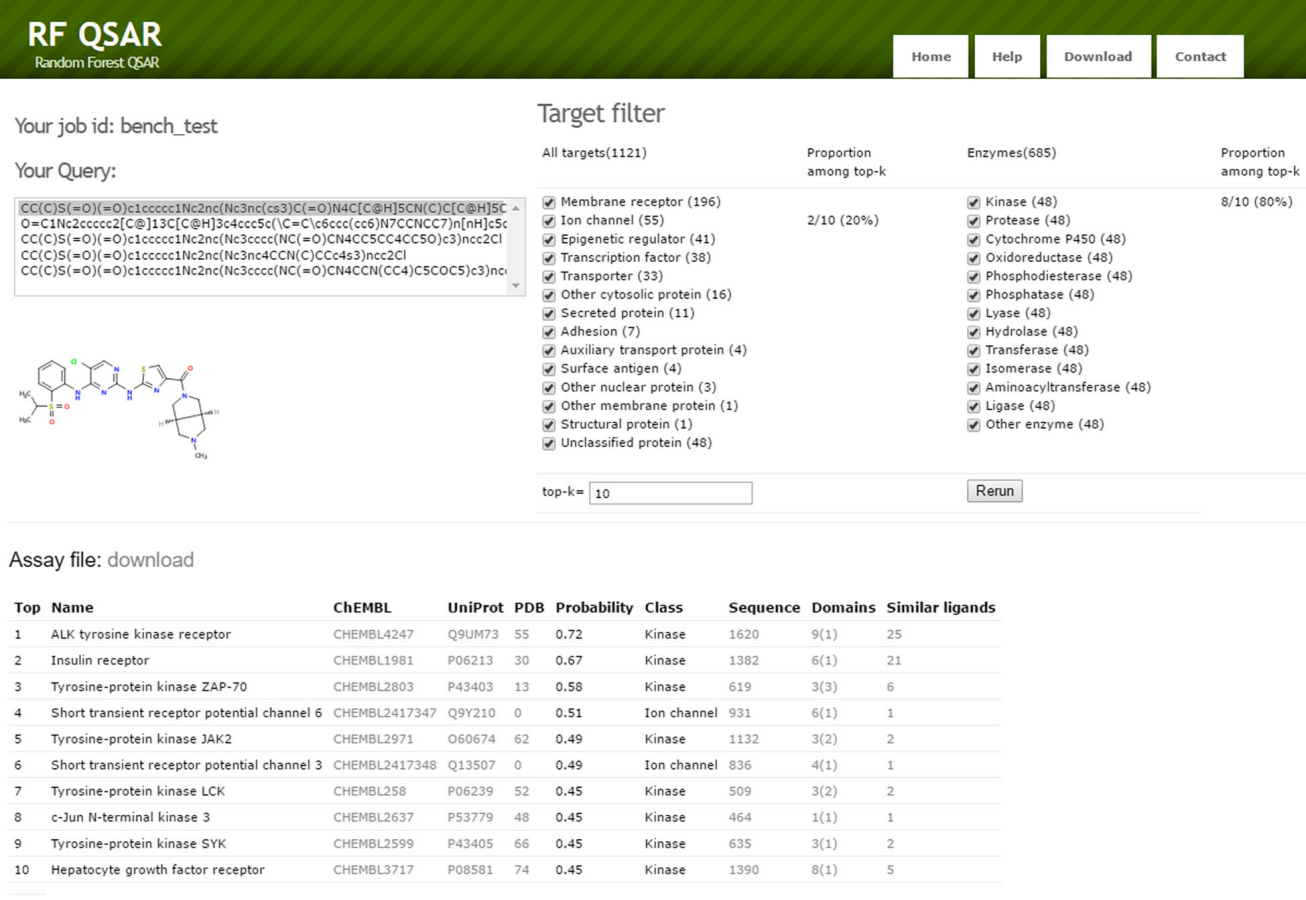
The result page of RF-QSAR web server. Query ligands to look over can be selected from the box. List of top-*k* targets and their information are provided in the table including name, ChEMBL ID, UniProt ID, PDB id, probability to be active, target class, sequence, domains, and ligands similar with the query from the target. Details about PDB id, sequence, domains, and similar ligands are linked by the numbers to other pages because the text is too long to write in the table. Users can re-rank the targets with different class filter and top-*k* threshold without repeating virtual screening. The virtual screening result also can be downloaded

We developed a target-fishing server named RF-QSAR [37]. Using RF-QSAR, users can identify targets of multiple query ligands at a time. Each ligand is assessed by 1121 target models and score matrix between ligands and targets are made. The score matrix is also converted to the probability matrix, where each cell indicates the probability of the ligand-target interaction being active. The matrix can be downloaded by link so that users can further utilize the score matrix for other researches. For example, scores from target models can be used as a profile of the ligand and the toxicity of the ligand can be predicted by the profile [20]. Server offers top-*k* targets ranked by the probability to interact with the ligand. The *k*-value and target classes to search can be determined by users according to the purpose of target-fishing. For top-ranked targets, information and cross references including Uniprot ID, target class, sequence, domains, and similar ligands are provided. The proportion of each target class of the ranked targets is also presented so that users can estimate the general target classes for a query ligand. Figure 10 shows the demonstration of RF-QSAR. In addition, we plan to add to the server several new functionalities such as searching preferred targets using protein sequence and highlighting common targets that are repeatedly found for different query ligands.

## Conclusions

We developed a ligand-based SAR model comprising 1121 individual target models trained with human bioactivity data retrieved from ChEMBL database using a random forest algorithm. The sampling method and parameters used for the data preprocess were carefully optimized by five-fold cross validation to maximize the recall rates for the top-rank targets. The active data of every target model were oversampled until the ratio of the number of inactive ligands to the number of active ligands was set to one. In addition, the inactive ligands similar to the active ligands with a Tc cutoff higher than 0.5 were excluded from the model-building process. Through this process, our model could overcome the imbalance between the classes or targets, and avoid ambiguity of inactive ligands. The resulting target models are available not only for predicting the activity of the ligands but for target fishing of a query ligand offering ranked target list. The performance of each target model was assessed by employing individual ROC curve and mean score, which showed its strength in distinguishing between the active and inactive ligands. The performance of the target ranking was validated using the recall rates of the top-*k* targets. Through the external validation, the recall rates were obtained as 67.6% for the top 1% targets and 73.9% for the top 3% targets. These results demonstrate that the performance obtained in this study is the highest, particularly for a relatively difficult test set having an average Tc similarity of 0.55 with the training set. The processes were validated using a unified scoring scheme, which was further fitted to the probability using an external dataset.

The web interface of RF-QSAR was designed to be user-friendly, offering intuitive result pages. Users can submit multiple query ligands and check the result at a time. The result page shows a ranked target list with estimated probability of interaction. Various information and cross references are provided for each target. One of the distinctive features of our site is filtering the targets in terms of their classes. Using this function, users can specify target classes to search or remove classes. Users can utilize our server for various purpose including target-fishing, ligand comparison, and profile building.

## Additional files

Additional file 1: MySQL codes for bioactivity extraction from ChEMBL database. Variable “molregno” from table “compound_structures” is identification code for ligands while variable “tid” from table “target_dictionary” is identification code for targets. (TXT 1 kb)

Additional file 2: Fitting model scores to the estimated probabilities. It contains mathematical expression used to fit a graph of log-scaled score versus estimated probability to the sigmoid function. (PDF 235 kb)

## Abbreviations

AUC: Area under curve
ECFP: Extended-connectivity fingerprints
ROC: Receiver-operating characteristic
SARs: Structure activity relationships
Tc: Tanimoto coefficient
