# Supplementary material for: Utilizing random Forest QSAR models with optimized parameters for target identification and its application to target-fishing server
Source: BMC Bioinformatics. 2017 Dec 28;18(Suppl 16):567. doi: 10.1186/s12859-017-1960-x (PMC5751401; doi:10.1186/s12859-017-1960-x)
Supplement: Supplementary file 2 — Fitting model scores to the estimated probabilities. It contains mathematical expression used to fit a graph of log-scaled score versus estimated probability to the sigmoid function. (PDF 235 kb) [file 12859_2017_1960_MOESM2_ESM.pdf]

When X = score and Y = estimated probability,

Let X^* = log(X).

Then, fit Y = f(X^*) to sigmoid function.

$$Y = \frac{1}{1 + e^{aX^* + b}} = \frac{1}{1 + e^{alogX + b}} = \frac{1}{1 + e^b \times X^a}$$

Fitted coefficients are a = -1.041 and b = -1.733.

Assuming a ~ -1,

$$Y = \frac{1}{1 + e^b \times X^a} \sim \frac{1}{1 + \frac{0.18}{X}} = \frac{X}{X + 0.18}$$

The values of the estimated probabilities range from 0 to 0.85 while the values of scores range from 0 to 1.
